# The role of tryptophan hydroxylase‐1 in metabolic dysfunction‐associated steatotic liver disease

**DOI:** 10.1002/ped4.70078

**Published:** 2026-08-31

**Authors:** Shuangzhen Jia, Xiaolin Ye, Zhaoxia Wang, Jie Wu

**Affiliations:** ^1^ Department of Gastroenterology, Hepatology and Nutrition Center, Beijing Children's Hospital, Capital Medical University National Center for Children's Health Beijing China; ^2^ Department of Gastroenterology Shenzhen Children's Hospital Shenzhen China

**Keywords:** Children, Metabolic dysfunction‐associated steatotic liver disease (MASLD), Parkin‐dependent mitophagy, Tryptophan hydroxylase‐1 (TPH1)

## Abstract

**Importance:**

Metabolic dysfunction‐associated steatotic liver disease (MASLD) is caused by dysregulated lipid metabolism, inflammation, and mitochondrial dysfunction. Given the rising burden of MASLD in children and adolescents, identifying experimentally tractable mechanisms relevant to pediatric diseases is of considerable interest. Tryptophan hydroxylase‐1 (TPH1), the rate‐limiting enzyme for peripheral serotonin synthesis, has been implicated in metabolic disorders, but its hepatic role in MASLD remains unclear.

**Objective:**

To investigate whether *Tph1* suppression alleviates lipid accumulation, mitochondrial dysfunction, and hepatocyte injury through changes associated with Parkin‐mediated mitophagy.

**Methods:**

MASLD was induced in young adult male C57BL/6J mice by a 12‐week high‐fat diet (HFD). At HFD onset, mice received a single tail‐vein injection of liver‐tropic AAV8‐shTph1 or the control AAV8. The HFD feeding was continued for 12 weeks until the end of the experiment. Hepatic histology, serum biochemistry, oxidative stress, inflammatory responses, apoptosis, and mitochondrial function were also evaluated. Mechanistic studies were conducted in palmitic acid/oleic acid‐treated AML12 hepatocytes using siRNA targeting *Tph1* and *Parkin*.

**Results:**

Hepatic TPH1 expression was elevated in MASLD mice. *Tph1* knockdown alleviated diet‐induced steatosis, reduced serum triglycerides, total cholesterol, low‐density lipoprotein cholesterol, and transaminase levels, suppressed oxidative stress, and attenuated inflammatory and fibrotic markers. *Tph1* silencing decreased hepatocyte apoptosis and restored mitochondrial function. Transmission electron microscopy and fractionated Western blotting analyses revealed increased Parkin‐associated mitophagy. In AML12 cells, siTph1 reduced lipid accumulation and apoptosis, whereas co‐silencing *Parkin* partially reversed these effects.

**Interpretation:**

Hepatic TPH1 is associated with MASLD progression and impaired Parkin‐mediated mitophagy. *Tph1* suppression may represent a potential therapeutic strategy for experimental MASLD, with possible translational relevance to pediatric MASLD, though direct clinical validation is still needed.

## Graphical abstract

Silencing hepatic tryptophan hydroxylase 1 (TPH1) alleviated diet‐induced steatotic liver disease by reducing lipid accumulation, oxidative stress, inflammation, fibrosis, and hepatocyte apoptosis. These protective effects were mediated, at least in part, through restoration of Parkin‐dependent mitochondrial autophagy, identifying hepatic TPH1 as a potential therapeutic target.

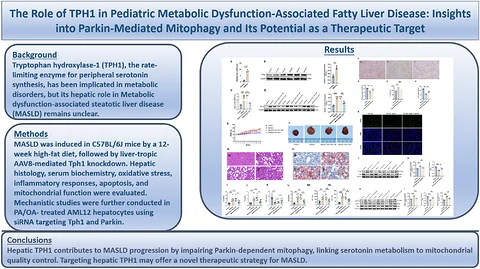

## INTRODUCTION

Metabolic dysfunction‐associated steatotic liver disease (MASLD), formerly known as non‐alcoholic fatty liver disease (NAFLD), is characterized by excessive hepatic lipid deposition accompanied by at least one metabolic abnormality.^1^ Unlike the traditional NAFLD definition, which excludes substantial alcohol intake as a prerequisite, the MASLD nomenclature emphasizes the central role of metabolic dysregulation in disease development, aligning more closely with the modern concept of the liver as a metabolic organ.^2^ Epidemiologically, the prevalence of MASLD is increasing at an alarming rate. A global analysis estimated that by 2021, the number of individuals with MASLD exceeded 1.26 billion worldwide, with an age‐standardized prevalence of approximately 15 000 per 100 000 population and an average annual increase of 0.7% since 1990.^3^ China is among the countries with the highest MASLD burden, and projections suggest that by 2030, the number of affected individuals may reach 314 million, with a prevalence of 22.2%.^3^ Hepatic steatosis, the earliest and most common lesion, may progress to metabolic‐associated steatohepatitis (MASH), fibrosis, cirrhosis, or hepatocellular carcinoma, making it a major cause of liver transplantation and liver‐related mortality.^4^ Moreover, MASLD is closely associated with multiple systemic disorders and is a significant risk factor for type 2 diabetes, metabolic syndrome, and cardiovascular disease. Notably, MASLD is no longer considered a disease in adulthood alone. Pediatric‐specific studies and clinical guidance indicate that steatotic liver disease is increasingly being recognized in children and adolescents, in parallel with the rising prevalence of pediatric obesity. In affected children, steatohepatitis and fibrosis may already be present in a subset of cases, and longitudinal cohort data suggest a persistent disease burden and adverse long‐term outcomes in some patients. Therefore, early identification and risk stratification in pediatric populations are clinically important.^5^, ^6^, ^7^, ^8^ Given its high prevalence and serious complications, MASLD represents a substantial public health, medical, and economic burden.

The pathogenesis of MASLD is multifactorial, involving genetic predisposition, insulin resistance, dysregulated lipid metabolism, inflammation, and oxidative stress.^9^, ^10^, ^11^ Mitochondria are highly dynamic organelles essential not only for ATP production but also for regulating ion homeostasis, particularly intracellular calcium, lipid, and cholesterol metabolism, redox balance, cell survival, and cell death. Increasing evidence indicates that mitochondrial dysfunction plays a pivotal role in MASLD progression. Damaged mitochondria fail to generate sufficient ATP to maintain hepatocyte homeostasis, leading to disruptions of lipid metabolism, glucose regulation, and protein synthesis. Mitochondrial injury exacerbates oxidative stress, impairs membrane potential, and promotes the release of pro‐apoptotic factors into the cytosol, ultimately triggering mitochondrial‐dependent hepatocyte death.^12^ Mitophagy, the selective degradation of damaged mitochondria, is essential for maintaining mitochondrial quality and function and has been recognized as a protective mechanism against MASLD progression.^13^, ^14^, ^15^ The Parkin‐mediated mitophagy pathway has attracted particular interest, as Parkin ubiquitinates dysfunctional mitochondria, facilitating their autophagic clearance and mitigating oxidative stress and inflammatory injury in hepatocytes.^16^, ^17^ Despite an increasing number of studies, the underlying mechanisms driving MASLD progression remain incompletely understood, and effective targeted therapies are lacking. Therefore, identifying key regulatory molecules and new therapeutic targets is crucial for MASLD prevention and treatment.

Tryptophan hydroxylase‐1 (TPH1), the rate‐limiting enzyme in peripheral serotonin (5‐hydroxytryptamine, 5‐HT) biosynthesis, is expressed not only in the nervous system but also in enterochromaffin cells, adipocytes, and other non‐neuronal tissues.^18^ As an important monoamine neurotransmitter, 5‐HT regulates both the central and peripheral physiological functions. In the liver, 5‐HT participates in the regulation of hemodynamics, metabolic processes, and immune responses, potentially influencing hepatic homeostasis and disease progression.^19^, ^20^, ^21^ Preliminary studies have shown that hepatic TPH1 expression is upregulated in models of glucocorticoid‐induced steatosis and insulin resistance.^22^, ^23^ These findings suggest that TPH1 may represent a potential therapeutic target for metabolic disorders such as obesity and fatty liver disease. However, the functional significance and mechanistic role of TPH1 in MASLD remain unclear and warrant further investigation. Experimental studies that identify mechanistic pathways potentially relevant to MASLD may help inform future translational and clinical research, although direct extrapolation to pediatric populations requires further validation.

## METHODS

### Ethical approval

All experimental procedures were approved by the Ethics Committee of Capital Medical University (Approval No.: AEEI‐2025‐405).

### Animals

Male C57BL/6J mice aged 6–8 weeks and weighing 18–22 g were purchased from SPF Biotechnology Co., Ltd. [Beijing, China; animal production license No. SCXK (Beijing) 2024‐0001]. Mice were housed under SPF conditions with controlled temperature (22–24°C), humidity (50%–60%), and a 12‐h light/dark cycle, with ad libitum access to food and water. Prior to the experiments, animals were acclimatized to the housing environment for 5 days and uniquely identified by ear tags to ensure health status monitoring.

### MASLD mouse model induction and adeno‐associated virus treatment

Mice were randomly assigned into four groups (*n* = 6 per group): (1) Control group: fed a standard chow diet; (2) MASLD group: fed a high‐fat diet (HFD) for 12 weeks; (3) MASLD + adeno‐associated virus (AAV)‐Tph1 negative control (NC) group: HFD plus tail‐vein injection of control AAV; and (4) MASLD + AAV‐Tph1 group: HFD plus tail‐vein injection of recombinant AAV8 carrying Tph1‐specific shRNA. HFD (D12492; Research Diets, Inc., New Brunswick, NJ, USA) contained approximately 60% fat, 20% protein, and 20% carbohydrates, with a caloric density of 5.24 kcal/g.

This study was designed as a preventive intervention protocol. After the acclimatization period, HFD feeding was initiated at week 0, and AAV was administered at the same time point by tail‐vein injection. Thus, viral intervention preceded the development of overt MASLD, and no fatty liver phenotype was present before AAV treatment. HFD feeding was continued for 12 weeks until the end of the experiment. The mice were monitored weekly for body weight, food intake, and general health. At the end of 12 weeks, MASLD development was confirmed by histological and biochemical assessments.

To knock down *Tph1* *in vivo*, recombinant AAV8 expressing shRNA targeting *Tph1* [AAV‐Tph1; 1 × 10^12^ vector genomes (vg) per mouse; packaged by GenePharma, Shanghai, China] was administered via tail‐vein injection in a total volume of 200 µL per mouse, diluted in phosphate‐buffered saline (PBS). The MASLD + AAV‐Tph1 NC group received an equal volume of empty vector virus at the same viral titer and total dose to control for vector‐related effects. The target sequences (5’–3’) of shRNAs harbored in the AAV vectors were as follows: shTph1, CACCCTGGCTTCAAAGACAAT; shTph1‐NC, TTCTCCGAACGTGTCACGT.

### Tissue collection

After 12 weeks of HFD feeding and 12 weeks after AAV administration, mice were fasted for approximately 12 h and anesthetized with isoflurane. Blood was collected via retro‐orbital puncture and centrifuged to obtain serum for biochemical analyses. Mice were sacrificed by cervical dislocation, and livers were perfused in situ with ice‐cold PBS via cardiac cannulation until the tissue color changed from dark red to pale, indicating complete blood removal. The livers were excised, trimmed of connective tissue and gallbladder, lightly blotted dry, and photographed on a calibrated sterile board. Liver weight was recorded using an analytical balance. The liver was divided into three sections for transmission electron microscopy (TEM), histology/immunohistochemistry (IHC), and molecular analyses.

### Cell culture and treatment

The mouse hepatocyte cell line AML12 (Shanghai FuHeng Biotechnology Co., Ltd.) was cultured in DMEM/F‐12 medium supplemented with 10% fetal bovine serum (FBS), 10 µg/mL insulin, 5.5 µg/mL transferrin, 5 ng/mL selenium, 40 ng/mL dexamethasone, 100 U/mL penicillin, and 100 µg/mL streptomycin in a humidified incubator at 37°C with 5% CO_2_. Cells were passaged when they reached 80%–90% confluence.

AML12 cells were transfected with Tph1‐specific siRNA (siTph1) or NC siRNA (siTph1‐NC) using Lipo6000 transfection reagent (Beyotime, China) according to the manufacturers' instructions. Briefly, cells were transfected at 70%–80% confluence and cultured for 24–48 h. The knockdown efficiency was confirmed by quantitative real‐time polymerase chain reaction (qRT‐PCR) and western blotting. For rescue experiments, Parkin siRNA was co‐transfected with siTph1 using the same protocol. The sequences of siRNAs used in this study are listed in Table S1.

### Lipid metabolic and liver function assays

Serum levels of alanine aminotransferase (ALT), aspartate aminotransferase (AST), triglycerides (TG), total cholesterol (TC), high‐density lipoprotein cholesterol (HDL‐C), and low‐density lipoprotein cholesterol (LDL‐C) were measured using an automated biochemical analyzer, according to the manufacturers' instructions.

### Biochemical analysis

Colorimetric assays were performed using commercial kits to measure hepatic superoxide dismutase (SOD) activity (BC0170, Solarbio), malondialdehyde (MDA) content (BC0025, Solarbio), reduced glutathione (GSH) levels (BC1175, Solarbio), and adenosine triphosphate (ATP) content (BC0300, Solarbio), following the manufacturers' protocols.

### Enzyme‐linked immunosorbent assay

Inflammatory cytokines in liver tissue were quantified using enzyme‐linked immunosorbent assay (ELISA) kits (Wuhan Hualian Keshi Biotechnology Co., Ltd.). Tumor necrosis factor‐α (TNF‐α; DRE30030), interleukin‐6 (IL‐6; DRE30027), and interleukin‐1β (IL‐1β; DRE30044) levels were measured according to the manufacturers' protocols.

### Histological and immunohistochemical analysis

Hematoxylin–eosin (H&E) staining was performed on paraffin‐embedded liver sections to evaluate hepatic morphology, including hepatocyte arrangement, nuclear appearance, steatosis, and inflammatory cell infiltration. Oil Red O staining was performed on frozen liver sections to visualize neutral lipid droplets and assess the degree of hepatic steatosis. Masson's trichrome staining was performed on paraffin sections to assess collagen deposition and the extent of hepatic fibrosis. All histological staining procedures were carried out following standard protocols, and images were acquired under a light microscope. Terminal deoxynucleotidyl transferase‐mediated dUTP nick‐end labeling (TUNEL) staining was performed on paraffin sections to localize and quantify hepatocyte apoptosis. For quantitative histological analysis, one consecutive coronal liver section from each mouse was used for pathological evaluation and image‐based quantification. Whole‐slide panoramic scanning was performed to capture the entire hepatic parenchymal region, covering the overall lobular architecture, rather than manually selecting local microscopic fields. During image analysis, non‐parenchymal regions, including the liver capsule, vascular lumina, tissue folds, and background debris, were excluded. All samples processed within the same batch were analyzed using identical thresholding and staining segmentation criteria, and microscope exposure, brightness, and contrast settings were kept constant. Positive staining area was quantified using a unified analysis workflow.

For IHC, paraffin sections were deparaffinized and rehydrated using a graded ethanol series. Sections were then incubated overnight at 4°C with primary antibodies against α‐SMA (ab7817; Abcam, 1:250) and Collagen I (ab34710; Abcam, 1:250). On the following day, sections were incubated with horseradish peroxidase (HRP)‐conjugated secondary antibodies at 37°C for 30 min. Color development was performed using DAB for approximately 5 min, followed by hematoxylin counterstaining. After dehydration, clearing in xylene, and mounting with neutral resin, stained slides were examined under a microscope. Positive staining areas were quantified using the ImageJ software to evaluate the degree of hepatic fibrosis. For cellular immunofluorescence analysis, three samples were analyzed per group, and at least six randomly selected non‐overlapping fields were acquired from each sample. The microscope illumination, exposure time, and image acquisition parameters were kept constant within the same experiment. Image segmentation was performed using a fixed uniform threshold, and nonspecific background fluorescence and field artifacts were excluded prior to quantification. Fluorescence intensity and the proportion of positive signal were quantified using the same analysis criteria across all groups.

### Transmission electron microscopy

TEM was employed to examine the ultrastructure of the hepatic mitochondria. Liver samples fixed in glutaraldehyde were post‐fixed in 1% osmium tetroxide, dehydrated using graded ethanol and acetone, and embedded in epoxy resin. Ultrathin sections (∼70 nm) were prepared and stained with 3% uranyl acetate for 20 min, followed by lead citrate for 10 min. Sections were observed using TEM (Hitachi HT7700, 80 kV), and the mitochondrial cristae structure, membrane integrity, and mitophagic structures were analyzed. Representative images were captured for both qualitative and quantitative assessments.

### RNA extraction and gene expression analysis

Total RNA from the cells and liver tissues was extracted using TRIzol reagent (M5102, Ncm Biotech). Reverse transcription was performed using the HiScript II Q RT SuperMix for qPCR (+gDNA wiper) kit (R223; Vazyme) according to the manufacturers' instructions. qRT‐PCR was conducted using ChamQ SYBR qPCR Master Mix (Q331‐03; Vazyme). Relative mRNA expression levels were calculated using the 2^−ΔΔCt^ method. Primer sequences used for qPCR are listed in Table S2.

### Isolation and detection of mitochondrial DNA by qRT‑PCR

Mitochondrial DNA (mtDNA) was isolated from liver tissues using a commercial mitochondrial DNA isolation kit (D2900; Solarbio), following the manufacturers' instructions. mtDNA levels were quantified by RT‐PCR, using primers targeting mtDNA‐encoded sequences. Relative mtDNA abundance was calculated using the 2^−ΔΔCt^ method and was normalized to *Actb* expression. Primer sequences are provided in Table S3.

### Western blotting analysis

Total protein from the liver tissues and cells was extracted using RIPA lysis buffer (P0013B; Beyotime). Mitochondrial proteins were isolated using a commercial mitochondrial isolation kit (C3601; Beyotime). Protein concentrations were determined using the BCA method (P0010; Beyotime). Equal amounts of protein (50 µg per lane) were denatured by boiling, separated on 12% SDS‐PAGE gels, and transferred onto PVDF membranes (IPVH00010; Millipore). Membranes were blocked with 5% non‐fat milk or BSA for 90 mins at room temperature and incubated overnight at 4°C with appropriately diluted primary antibodies, including TPH1 (29904‐1‐AP; Proteintech, 1:500), α‐SMA (ab7817; Abcam, 1:2000), Collagen I (ab34710, Abcam, 1:1000), LC3 (14600‐1‐AP; Proteintech, 1:1000), Parkin (14060‐1‐AP; Proteintech, 1:1000), P62 (18420‐1‐AP; Proteintech, 1:1000), cleaved‐caspase‐3 (68773‐1‐Ig; Proteintech, 1:5000), BAX (50599‐2‐Ig; Proteintech, 1:2000), cytochrome c (cyt‐c, Ab133504; Abcam, 1:5000), GAPDH (60004‐1‐Ig; Proteintech, 1:10000), β‐actin (66009‐1‐Ig; Proteintech, 1:5000), VDAC1 (55259‐1‐AP; Proteintech, 1:1000), and COXIV (11242‐1‐AP; Proteintech, 1:1000).

Following primary incubation, membranes were washed with Tris‐buffered saline with Tween 20 (TBST) and incubated with HRP‐conjugated secondary antibodies at room temperature for 120 min. After a second TBST wash, signals were developed using an ECL chemiluminescent substrate (180–501, Tanon) and imaged accordingly. Band intensities were quantified using ImageJ software. The ratio of the target protein band to GAPDH or β‐actin was used to determine relative protein expression levels.

### MTT assay

Cell viability was determined using an MTT assay kit (BS186; Biosharp, China) according to the manufacturers' instructions. Absorbance was measured at 490 nm using a microplate reader.

### JC‐1 staining

Mitochondrial membrane potential (ΔΨm) was evaluated using a JC‐1 Mitochondrial Membrane Potential Assay Kit (M8650; Solarbio, China). Following treatment, AML12 cells were incubated with JC‐1 staining solution at 37°C for 20 min according to the manufacturers' instructions. Cells were then washed with JC‐1 buffer and observed under a fluorescence microscope. The ratio of red to green fluorescence was used to assess changes in mitochondrial membrane potential.

### MitoSOX Red staining

Mitochondrial reactive oxygen species (mtROS) levels were detected using MitoSOX Red Mitochondrial Superoxide Indicator (M36008; Invitrogen, USA). AML12 cells were incubated with 5 µmol/L MitoSOX Red working solution at 37°C for 10 min in the dark. After washing with PBS, fluorescence images were captured using a fluorescence microscope, and fluorescence intensity was quantified using ImageJ software.

### MitoTracker and LC3 co‐localization immunofluorescence

Cells were stained with MitoTracker Deep Red FM (M7512; Invitrogen, USA), followed by immunofluorescence staining for LC3. Images were acquired using a confocal laser scanning microscope, and LC3‐mitochondria co‐localization was analyzed using ImageJ software.

### Statistical analysis

All data are presented as mean ± standard deviation (mean ± SD). Statistical analyses and graph preparation were performed using GraphPad Prism 9.0. Differences between the two groups were assessed using a two‐tailed unpaired Student's *t*‐test. Multiple group comparisons were analyzed using one‐way analysis of variance followed by Tukey's post‐hoc test. A *P*‐value < 0.05 was considered statistically significant.

## RESULTS

### *Tph1* knockdown alleviates hepatic injury and lipid metabolic disorders in MASLD mice

To confirm the successful establishment of the MASLD model, mice were fed HFD for 12 weeks and evaluated phenotypically and biochemically. Body weight monitoring showed a progressive increase in HFD mice compared with controls from week 4 onward, with a significant difference at week 12 (Figure S1A). Histological examination revealed marked hepatocyte swelling, vacuolar degeneration, and abundant lipid droplet accumulation in the livers of HFD‐fed mice (Figure S1B). Oil Red O staining confirmed extensive lipid deposition (Figure S1B). Serum biochemical analysis showed elevated ALT, AST, TG, TC, and LDL‐C levels, and decreased HDL‐C levels in HFD mice (Figure S1C–H), indicating hepatocellular injury and dysregulation of lipid metabolism. Collectively, these findings validated the successful generation of a MASLD mouse model.

**FIGURE 1:**
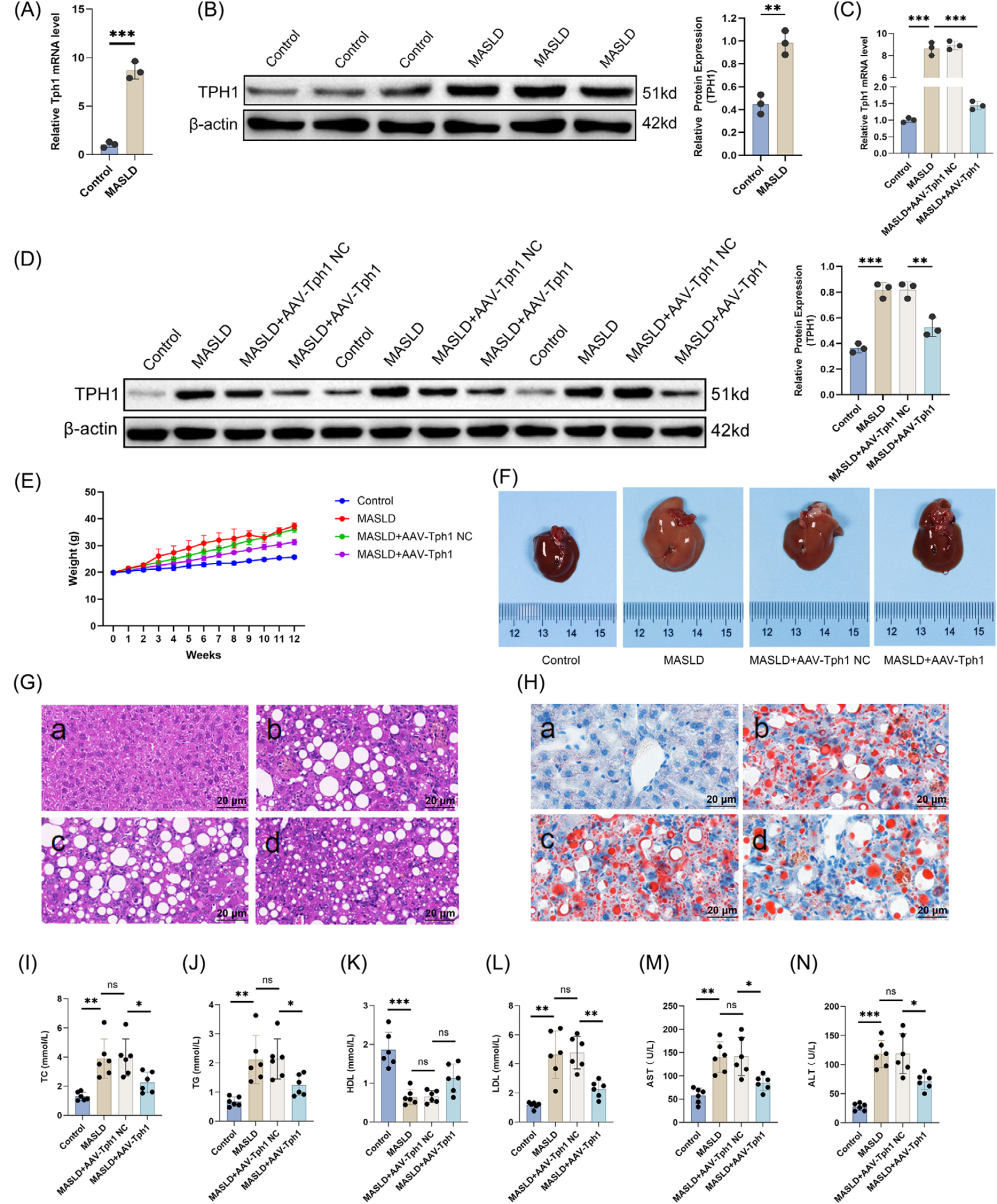
Knockdown of *Tph1* alleviates hepatic injury and steatosis in MASLD mice. (A, B) *Tph1* expression in liver tissue of control and MASLD mice was assessed by qPCR and Western blotting, showing upregulation in MASLD (*n* = 3 per group). (C, D) Validation of *Tph1* knockdown efficiency *in vivo*. *Tph1* mRNA and protein levels were significantly reduced in MASLD + AAV‐Tph1 mice compared to AAV‐NC controls (*n* = 3 per group). (E) Body weight changes during 12 weeks of high‐fat diet feeding. (F) Representative gross morphology of the liver. (G) Representative H&E staining images of four groups: (a) Control, (b) MASLD, (c) MASLD + AAV‐Tph1 NC, and (d) MASLD + AAV‐Tph1. Scale bar = 20 µm. (H) Representative Oil Red O staining images of four groups: (a) Control, (b) MASLD, (c) MASLD + AAV‐Tph1 NC, and (d) MASLD + AAV‐Tph1. Scale bar = 20 µm. (I–N) The levels of TG, TC, LDL‐C, HDL‐C, AST, and ALT in the serum (*n* = 6 per group). Data are presented as mean ± SD. ^*^ *P* < 0.05; ^**^ *P* < 0.01; ^***^ *P* < 0.001. MASLD, metabolic dysfunction‐associated steatotic liver disease; AAV, adeno‐associated virus; NC, negative control; TG, triglycerides; TC, total cholesterol; LDL‐C, low‐density lipoprotein cholesterol; HDL‐C, high‐density lipoprotein cholesterol; AST, aspartate aminotransferase; ALT, alanine aminotransferase.

To investigate the role of TPH1 in MASLD, qRT‐PCR and western blotting were performed to examine TPH1 expression in the liver tissues. Both *Tph1* mRNA and TPH1 protein levels were significantly increased in MASLD mice relative to controls (Figure 1A,B), suggesting that TPH1 upregulation may contribute to enhanced hepatic lipid metabolism disorder and inflammation. To explore its functional role, *Tph1* was knocked down specifically in the liver using AAV8. We validated the knockdown efficiency of AAV‐Tph1 using qPCR and Western blotting. *Tph1* mRNA expression was significantly decreased in liver tissues of MASLD mice treated with AAV‐Tph1 compared to AAV‐NC (Figure 1C), and protein expression levels were also markedly reduced (Figure 1D), confirming successful *in vivo* silencing of *Tph1*.

After *Tph1* knockdown, HFD‐induced weight gain was markedly attenuated compared with untreated MASLD mice (Figure 1E). Gross morphology showed enlarged, yellowish livers in MASLD mice, while AAV‐Tph1 treatment improved coloration and reduced liver size (Figure 1F). H&E staining revealed severe hepatocyte ballooning, steatosis, and inflammatory infiltration in MASLD livers, all of which were markedly improved following *Tph1* knockdown (Figure 1G). Oil Red O staining confirmed a significant reduction in neutral lipid accumulation (Figure 1H). Serum lipid analysis showed that *Tph1* knockdown effectively reduced TG, TC, and LDL‐C levels and restored HDL‐C levels (Figure 1I–L). Meanwhile, elevated ALT and AST levels in MASLD mice were markedly lowered after *Tph1* knockdown (Figure 1M,N). Collectively, these findings demonstrate that reducing TPH1 expression significantly attenuates hepatic steatosis, lipid accumulation, and liver injury in MASLD mice.

### *Tph1* knockdown mitigates hepatic oxidative stress, inflammation, and fibrosis in MASLD mice

**FIGURE 2:**
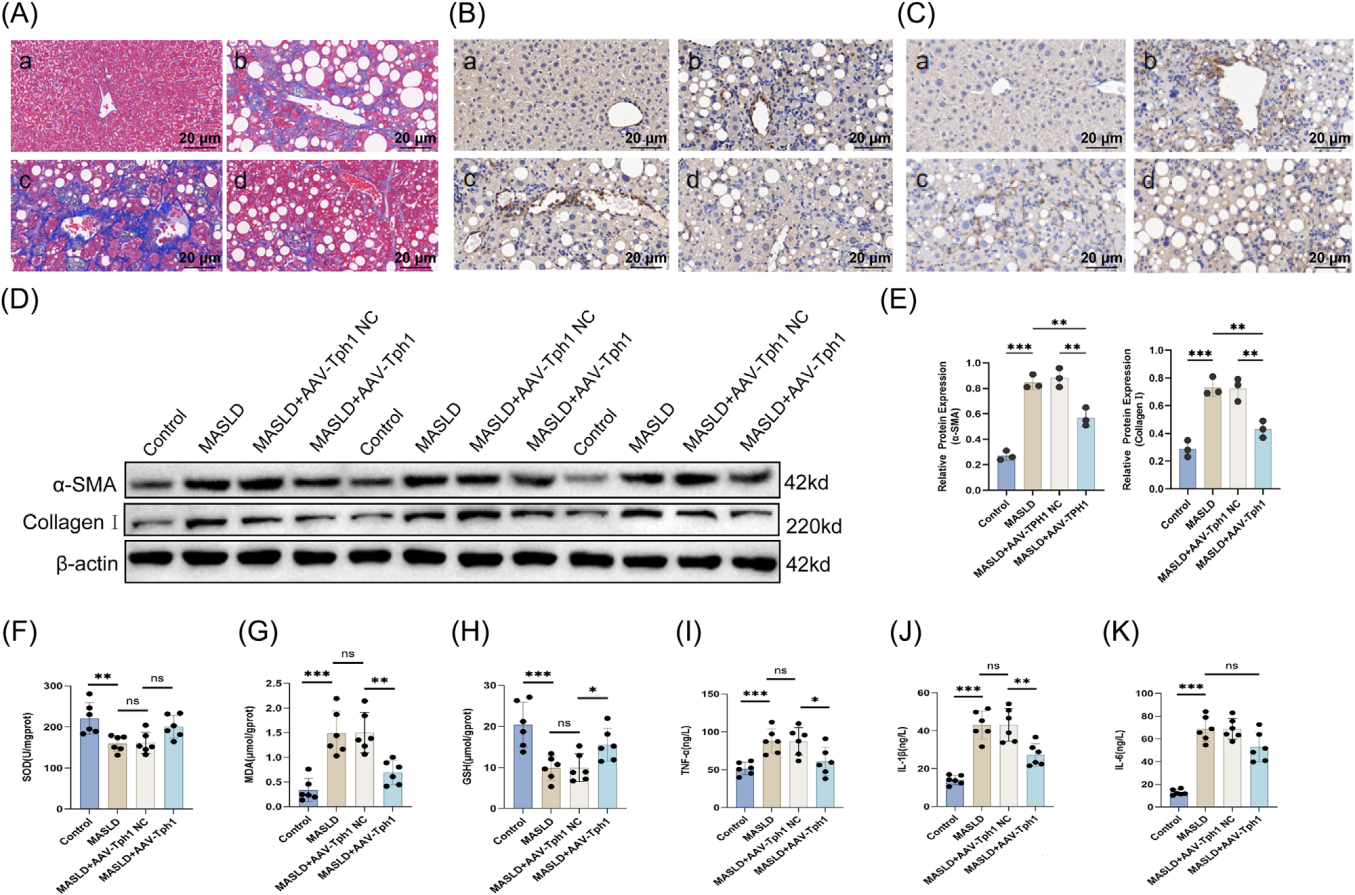
Knockdown of *Tph1* alleviates hepatic oxidative stress, inflammation, and fibrosis in MASLD mice. (A) Representative Masson staining images of four groups: (a) Control, (b) MASLD, (c) MASLD + AAV‐Tph1 NC, and (d) MASLD + AAV‐Tph1. Scale bar = 20 µm. (B, C) Immunohistochemical staining of α‐SMA and Collagen I in liver sections of four groups: (a) Control, (b) MASLD, (c) MASLD + AAV‐Tph1 NC, and (d) MASLD + AAV‐Tph1. Scale bar = 20 µm. (D, E) Western blotting analysis and quantification of α‐SMA and Collagen I protein expression in liver tissue (*n* = 3 per group). (F–K) The levels of SOD, MDA, GSH, TNF‐α, IL‐1β, and IL‐6 in liver tissue (*n* = 6 per group). Data are presented as mean ± SD. ^*^ *P* < 0.05; ^**^ *P* < 0.01; ^***^ *P* < 0.001. MASLD, metabolic dysfunction‐associated steatotic liver disease; AAV, adeno‐associated virus; NC, negative control; SOD, superoxide dismutase; MDA, malondialdehyde; GSH, glutathione; TNF, tumor necrosis factor; IL, interleukin.

Oxidative stress, inflammation, and fibrosis are hallmark pathological features of MASLD.^24^ To further evaluate the impact of *Tph1* knockdown on liver pathology, we examined indicators of fibrosis, oxidative stress, and inflammation. Masson staining revealed substantial collagen deposition in MASLD mice, while fibrosis was significantly reduced in the AAV‐Tph1 group (Figure 2A). IHC showed increased expression of α‐SMA and Collagen I in MASLD livers, whereas *Tph1* knockdown markedly diminished their positive staining (Figure 2B,C). Western blotting results further confirmed the downregulation of α‐SMA and Collagen I after AAV‐Tph1 treatment (Figure 2D,E), indicating attenuated fibrosis. Assessment of oxidative stress markers showed that SOD and reduced GSH levels were decreased, and MDA levels were elevated in MASLD mice (Figure 2F–H), indicating aggravated oxidative stress. *Tph1* knockdown significantly restored antioxidant capacity and reduced lipid peroxidation. Furthermore, ELISA results demonstrated elevated levels of TNF‐α, IL‐1β, and IL‐6 in MASLD liver tissues, whereas these inflammatory cytokines were significantly reduced after *Tph1* suppression (Figure 2I–K). Collectively, these findings suggest that *Tph1* knockdown attenuates oxidative stress, inflammation, and fibrosis, thereby slowing the pathological progression of MASLD.

### *Tph1* knockdown attenuates hepatocyte apoptosis and improves mitochondrial function in MASLD mice

**FIGURE 3:**
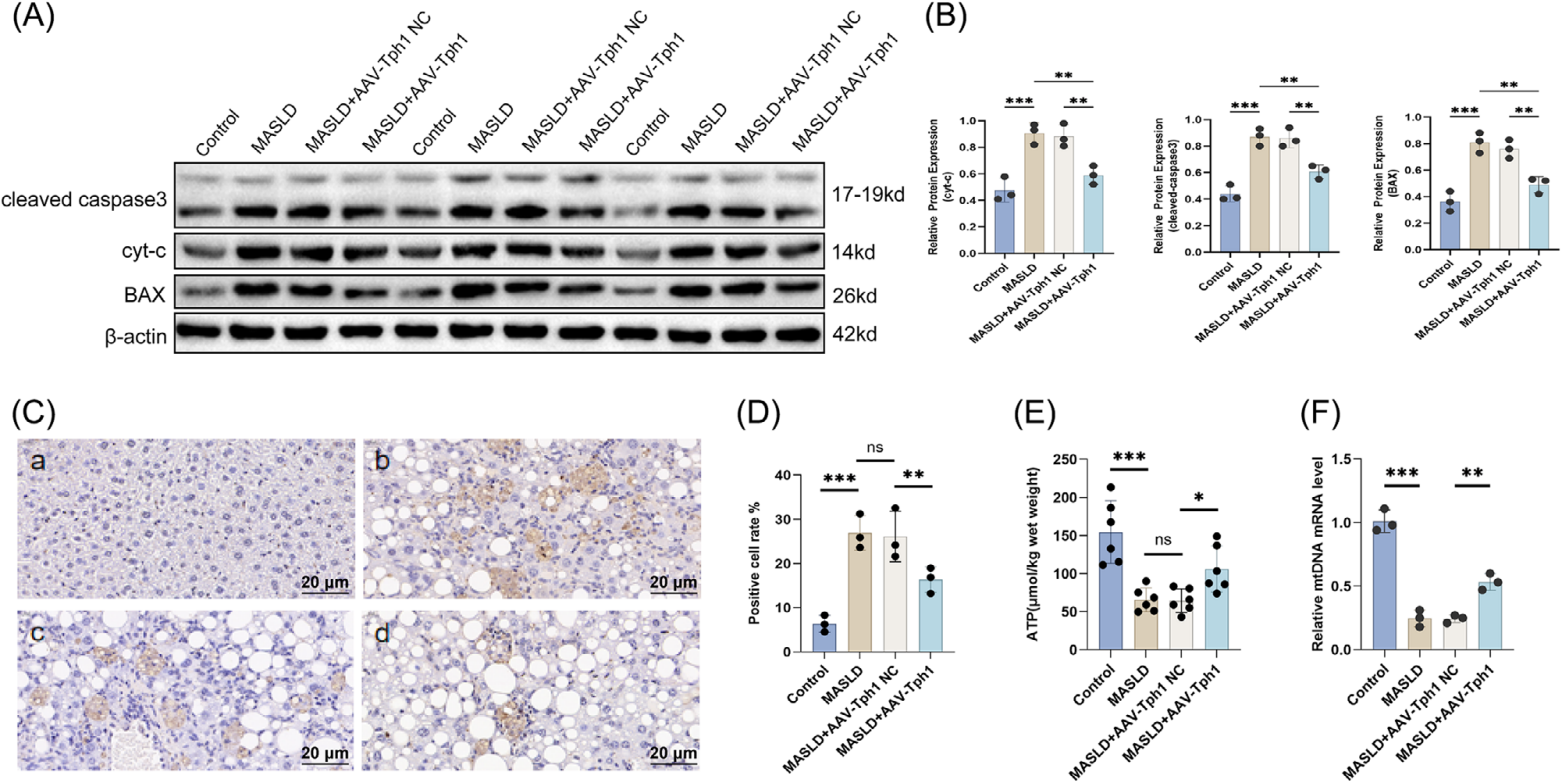
Knockdown of *Tph1* attenuates hepatocyte apoptosis and improves mitochondrial function in MASLD mice. (A, B) Western blotting analysis and quantification of cyt‐c, cleaved‐caspase‐3, and BAX protein expression in liver tissue (*n* = 3 per group). (C) Representative TUNEL staining images of four groups: (a) Control, (b) MASLD, (c) MASLD + AAV‐Tph1 NC, and (d) MASLD + AAV‐Tph1. Scale bar = 20 µm. (D) Quantitative analysis of apoptotic hepatocytes. (E) The levels of ATP in liver tissue (*n* = 6 per group). (F) qPCR quantification of mitochondrial DNA (mtDNA) expression in liver tissue (*n* = 3 per group). Data are presented as mean ± SD. ^*^ *P* < 0.05; ^**^ *P* < 0.01; ^***^ *P* < 0.001. MASLD, metabolic dysfunction‐associated steatotic liver disease; AAV, adeno‐associated virus; NC, negative control.

To further investigate the regulatory role of TPH1 in hepatocyte mitochondrial function and apoptosis in MASLD mice, we examined changes in relevant molecular markers. Western blotting analysis showed that, compared with the control group, MASLD mice exhibited markedly increased expression of cyt‐c, cleaved caspase‐3 (c‐caspase‐3), and the pro‐apoptotic protein BAX. These elevated levels were significantly reduced following *Tph1* knockdown (Figure 3A,B), indicating that *Tph1* suppression markedly alleviates hepatocyte apoptosis. Consistent with these findings, TUNEL staining revealed a substantial increase in TUNEL‐positive cells in MASLD liver tissues, whereas the number of apoptotic cells was significantly reduced after AAV‐Tph1 treatment (Figure 3C,D). Together, these data demonstrate that *Tph1* knockdown effectively inhibits hepatocyte apoptosis in MASLD mice.

To evaluate mitochondrial function, hepatic ATP levels and mtDNA content were assessed. MASLD mice exhibited a pronounced reduction in ATP production, whereas AAV‐Tph1 treatment significantly restored ATP levels (Figure 3E). qPCR analysis further revealed a significant decrease in hepatic mtDNA abundance in MASLD mice, while *Tph1* knockdown markedly increased mtDNA copy number (Figure 3F). Collectively, these findings indicate that *Tph1* silencing effectively alleviates MASLD‐induced hepatocyte apoptosis and improves mitochondrial function and energy metabolism.

### *Tph1* knockdown restores Parkin‐mediated mitophagy and ameliorates liver injury

**FIGURE 4:**
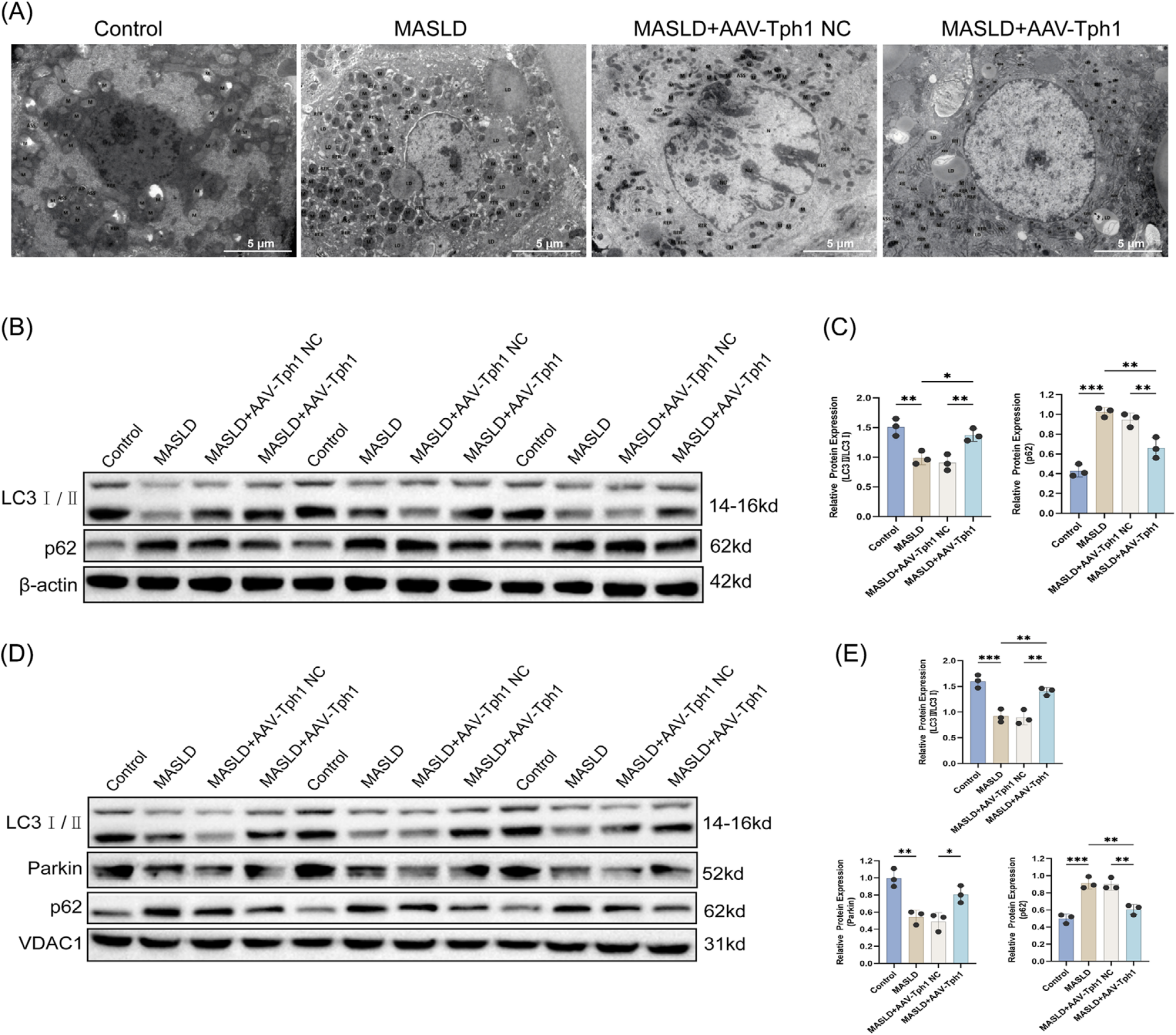
Knockdown of *Tph1* activates Parkin‐mediated mitophagy and alleviates hepatic injury in MASLD mice. (A) TEM images showing mitochondrial ultrastructure in liver tissue. Scale bar = 5 µm (B, C) Western blotting analysis and quantification of LC3 and p62 expression in total liver lysates (*n* = 3 per group). (D, E) Western blotting analysis and quantification of LC3, Parkin, and p62 protein levels in mitochondrial fractions (*n* = 3 per group). Data are presented as mean ± SD. ^*^ *P* < 0.05; ^**^ *P* < 0.01; ^***^ *P* < 0.001. TEM, transmission electron microscopy; MASLD, metabolic dysfunction‐associated steatotic liver disease; AAV, adeno‐associated virus; NC, negative control.

Normal mitophagy plays a protective role in MASLD and various other liver diseases.^25^ To further elucidate the involvement of TPH1 in mitochondrial quality control in MASLD, we evaluated mitophagy using TEM and expression of mitophagy‐related proteins. TEM analysis showed that the hepatocytes in the control group exhibited intact cellular structures, well‐organized mitochondrial cristae, and a small number of autophagosomes, reflecting basal autophagic activity. In contrast, MASLD mice displayed swollen hepatocytes, disrupted and swollen mitochondrial cristae, and a marked reduction in autophagosomes, indicating impaired mitophagy. Notably, in AAV‐Tph1‐treated mice, mitochondrial morphology was largely restored, and the numbers of autolysosomes and double‐membrane autophagosomes were significantly increased, suggesting increased mitophagy‐related activity (Figure 4A). Western blotting results further supported these observations. In total protein extracts, the MASLD group exhibited a decreased LC3‐II/I ratio and significant accumulation of p62, suggesting altered autophagy‐related activity; these abnormalities were reversed by *Tph1* knockdown, which increased LC3‐II/I and reduced p62 (Figure 4B,C). In mitochondrial protein fractions, LC3 and Parkin levels were reduced while p62 was elevated in MASLD mice, suggesting suppressed Parkin‐mediated mitophagy. *Tph1* knockdown significantly increased LC3 and Parkin expression and reduced p62 accumulation (Figure 4D,E). Together, these findings indicate that *Tph1* knockdown associates with changes suggestive of enhanced Parkin‐mediated mitophagy and attenuation of MASLD‐induced hepatic injury. However, they fail to definitively distinguish true mitophagy activation from altered autophagic flux, or to define the direct link between TPH1 and Parkin regulation.

### Inhibition of *Tph1* alleviates free fatty acid‐induced lipid accumulation and hepatocyte apoptosis

**FIGURE 5:**
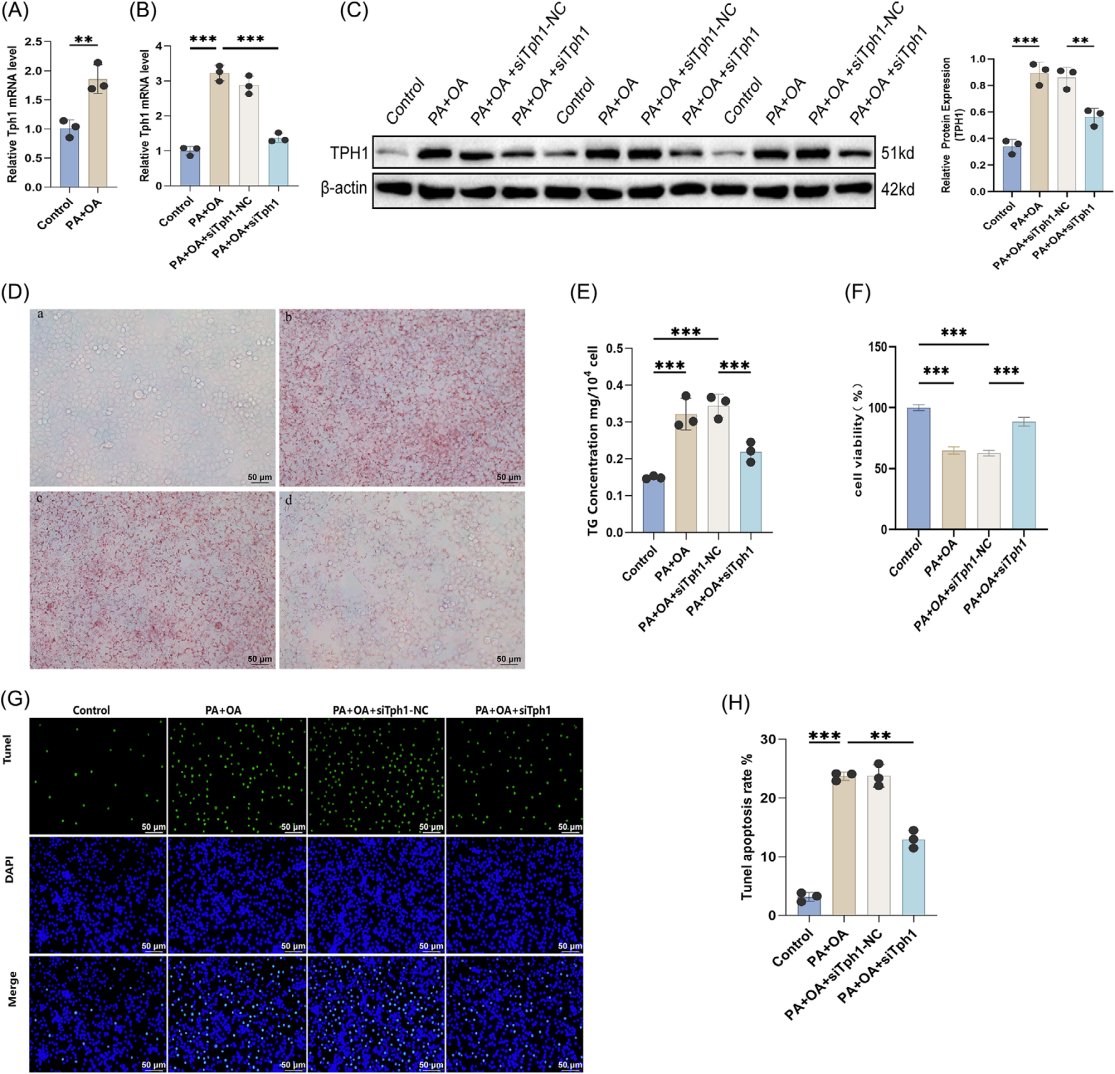
Inhibition of *Tph1* alleviated FFA‐induced lipid accumulation and apoptosis in AML12 cells. (A) The mRNA expression of *Tph1* was detected by qPCR after treatment with PA (0.25 mM) + OA (0.125 mM) for 24 h (*n* = 3 per group). (B, C) Validation of *Tph1* knockdown efficiency. qPCR and Western blotting analysis confirmed significantly decreased *Tph1* mRNA and TPH1 protein expression in the PA + OA + siTph1 group compared with the PA + OA + siTph1‐NC group Cell (*n* = 3 per group). (D) Lipid accumulation was assessed by Oil Red O staining. Representative images of four groups are shown: (a) control, (b) PA+OA, (c) PA + OA + siTph1‐NC, and (d) PA + OA + siTph1. Scale bar = 50 µm. (E) Quantification of intracellular triglyceride (TG) content (*n* = 3 per group). (F) Cell viability was evaluated by MTT assay. (G) Representative TUNEL fluorescence images of each treatment group. Scale bar = 50 µm. (H) Quantification of apoptotic cell percentage using TUNEL staining (*n* = 3 per group). Data are expressed as mean ± SD. ^**^ *P* < 0.01; ^***^ *P* < 0.001. NC, negative control; OA, oleic acid; PA, palmitic acid; FFA, free fatty acid.

To further validate the role and mechanism of TPH1 in the progression of MASLD, we established an *in vitro* model of AML12 hepatocytes induced by free fatty acid (FFA) [palmitic acid (PA) + oleic acid (OA)] and performed *Tph1* knockdown using siRNA. qPCR analysis showed that PA + OA treatment markedly increased *Tph1* mRNA expression compared with the control group (Figure 5A). To examine the effect of *Tph1* silencing, AML12 cells were transfected with *Tph1* siRNA or an NC. The efficiency of *Tph1* knockdown was validated by both qPCR and Western blotting. *Tph1* mRNA (Figure 5B) and protein (Figure 5C) levels were significantly reduced in the siTph1 group compared to the control, confirming effective gene silencing. Oil Red O staining revealed extensive intracellular lipid droplet accumulation following PA + OA exposure, whereas *Tph1* silencing significantly reduced lipid droplet area, indicating alleviated lipid deposition (Figure 5D). Consistent with this, intracellular TG content was significantly lower in the siTph1 group than in the PA + OA and siTph1 NC groups (Figure 5E). Functionally, MTT assays showed that PA + OA markedly reduced AML12 cell viability, whereas *Tph1* knockdown significantly restored proliferative capacity (Figure 5F). TUNEL staining demonstrated a substantial increase in apoptotic cells upon PA + OA exposure, while siTph1 markedly reduced TUNEL‐positive cells (Figure 5G), and quantitative analysis confirmed a significant decline in apoptosis rate (Figure 5H). Collectively, these results indicate that TPH1 promotes lipid accumulation and apoptosis during MASLD progression, whereas its inhibition significantly attenuates FFA‐induced lipotoxic injury.

### Inhibition of *Tph1* improves mitochondrial function in hepatocytes

**FIGURE 6:**
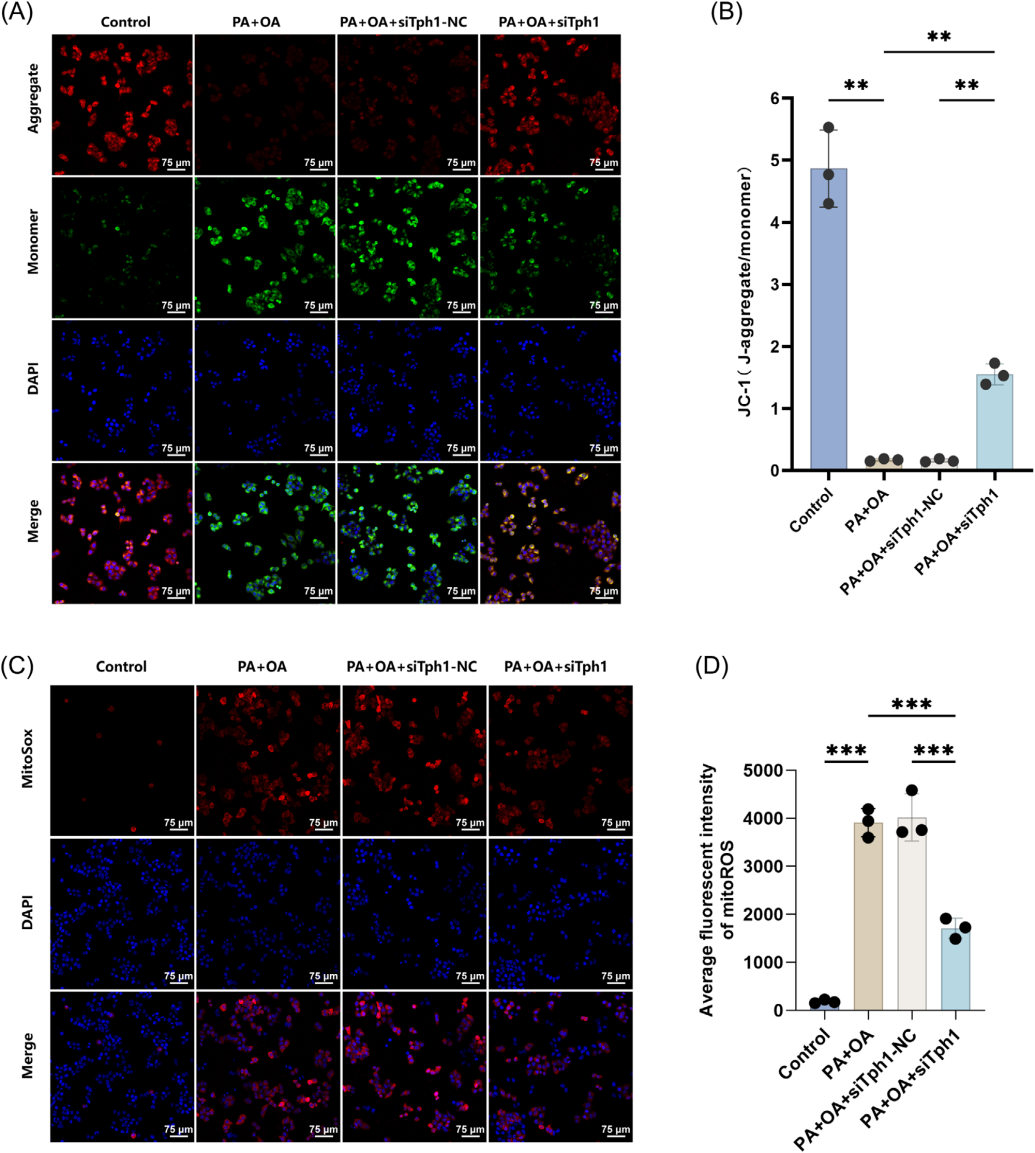
*Tph1* inhibition improved mitochondrial function in FFA‐treated AML12 cells. (A) Mitochondrial membrane potential was visualized by JC‐1 staining. Scale bar = 75 µm. (B) Quantification of the red/green fluorescence intensity ratio to assess mitochondrial membrane potential (*n* = 3 per group). (C) Mitochondrial reactive oxygen species were detected using MitoSOX Red staining. Scale bar = 75 µm. (D) Quantification of MitoSOX fluorescence intensity (*n* = 3 per group). Data are expressed as mean ± SD. ^**^ *P* < 0.01; ^***^ *P* < 0.001. NC, negative control; OA, oleic acid; PA, palmitic acid; FFA, free fatty acid.

To investigate the regulatory role of TPH1 in hepatocyte mitochondrial function, we assessed mitochondrial membrane potential (ΔΨm) and oxidative stress levels. JC‐1 staining demonstrated that PA + OA treatment markedly decreased the red/green fluorescence ratio, indicating loss of ΔΨm; however, *Tph1* knockdown significantly restored the red/green ratio, suggesting improved mitochondrial membrane potential (Figure 6A,B). In addition, mitochondrial ROS levels were examined using the MitoSOX Red probe. PA + OA treatment markedly increased mitochondrial ROS accumulation, whereas *Tph1* silencing significantly reduced ROS intensity (Figure 6C,D). These findings indicate that *Tph1* inhibition effectively improves FFA‐induced mitochondrial dysfunction by restoring membrane potential and reducing oxidative stress.

### Inhibition of *Tph1* activates mitophagy in hepatocytes

**FIGURE 7:**
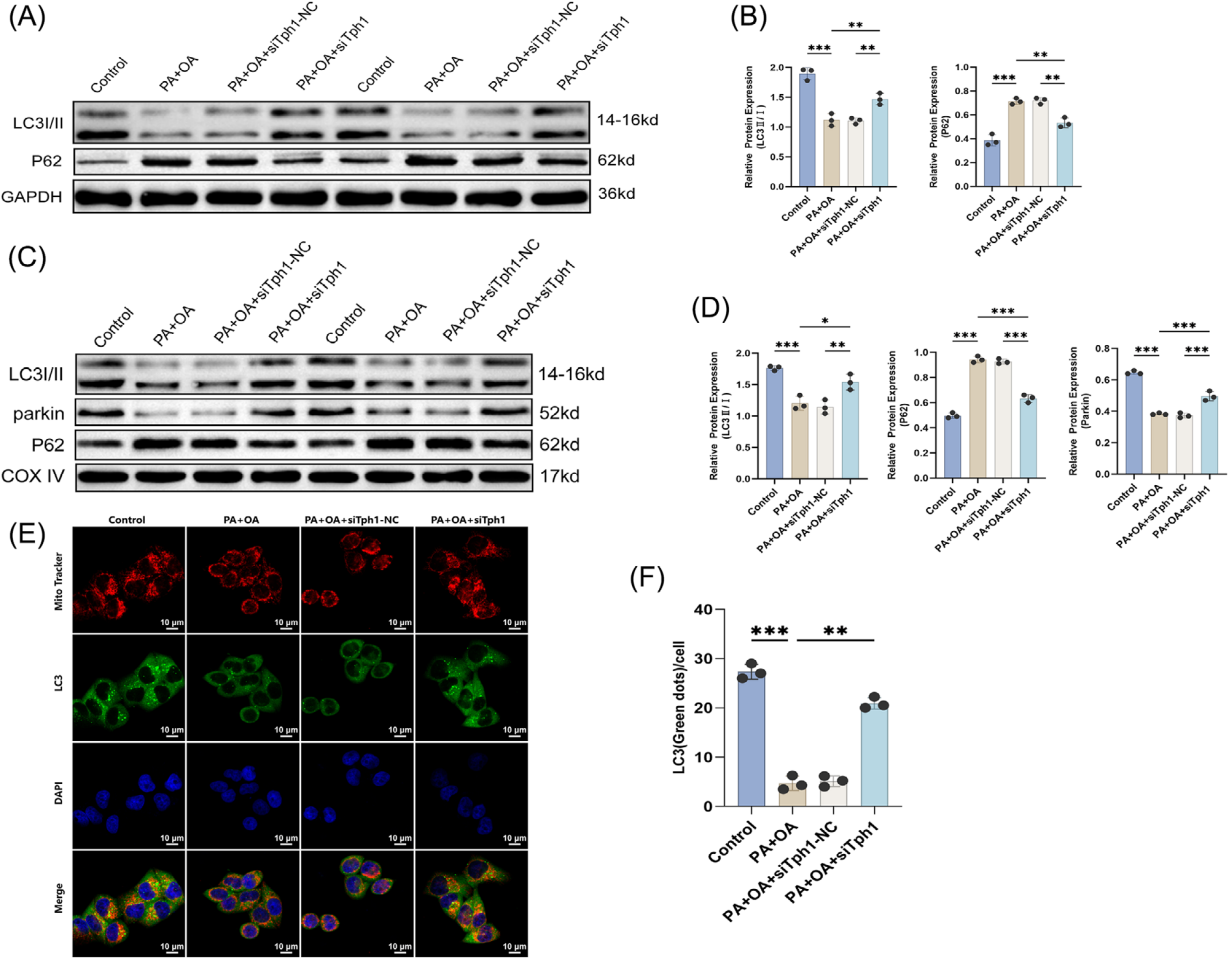
Inhibition of *Tph1* activated mitochondrial autophagy in AML12 cells. (A, B) Western blotting analysis and quantification of LC3‐II/I ratio and p62 levels in total cellular lysates (*n* = 3 per group). (C, D) Western blotting analysis and quantification of mitochondrial LC3, Parkin, and p62 protein expression (*n* = 3 per group). (E) Representative immunofluorescence images showing colocalization of LC3 puncta with mitochondria labeled by MitoTracker Deep Red. Scale bar = 10 µm. (F) Quantification of LC3 puncta per cell across treatment groups (*n* = 3 per group). Data are expressed as mean ± SD. ^*^ *P* < 0.05; ^**^ *P* < 0.01; ^***^ *P* < 0.001. NC, negative control; OA, oleic acid; PA, palmitic acid.

To determine whether TPH1 regulates mitophagy in hepatocytes, we examined autophagy‐related protein expression in total and mitochondrial protein fractions. Western blotting analysis revealed that PA + OA treatment reduced the LC3‐II/I ratio and caused substantial p62 accumulation, suggesting altered autophagy‐related activity. In contrast, *Tph1* knockdown significantly increased LC3‐II/I and reduced p62 expression, which was consistent with enhanced autophagy‐related signaling (Figure 7A,B). In mitochondrial fractions, *Tph1* silencing markedly increased Parkin levels, accompanied by upregulation of LC3‐II/I and downregulation of p62 (Figure 7C,D), suggesting enhanced Parkin‐associated mitophagy. To visualize mitophagy, MitoTracker Deep Red and LC3 co‐localization immunofluorescence was performed. PA + OA treatment resulted in weak LC3–mitochondria co‐localization signals, whereas *Tph1* knockdown markedly enhanced co‐localization intensity (Figure 7E). Quantification revealed a significant increase in LC3‐positive puncta following *Tph1* inhibition (Figure 7F). These results support an association between *Tph1* depletion and mitophagy‐related changes consistent with enhanced activity in hepatocytes, but they do not confirm true mitophagic flux activation, and do not clarify how TPH1 directly regulates Parkin.

### TPH1 promotes MASLD progression through Parkin‐mediated mitophagy dysregulation

**FIGURE 8:**
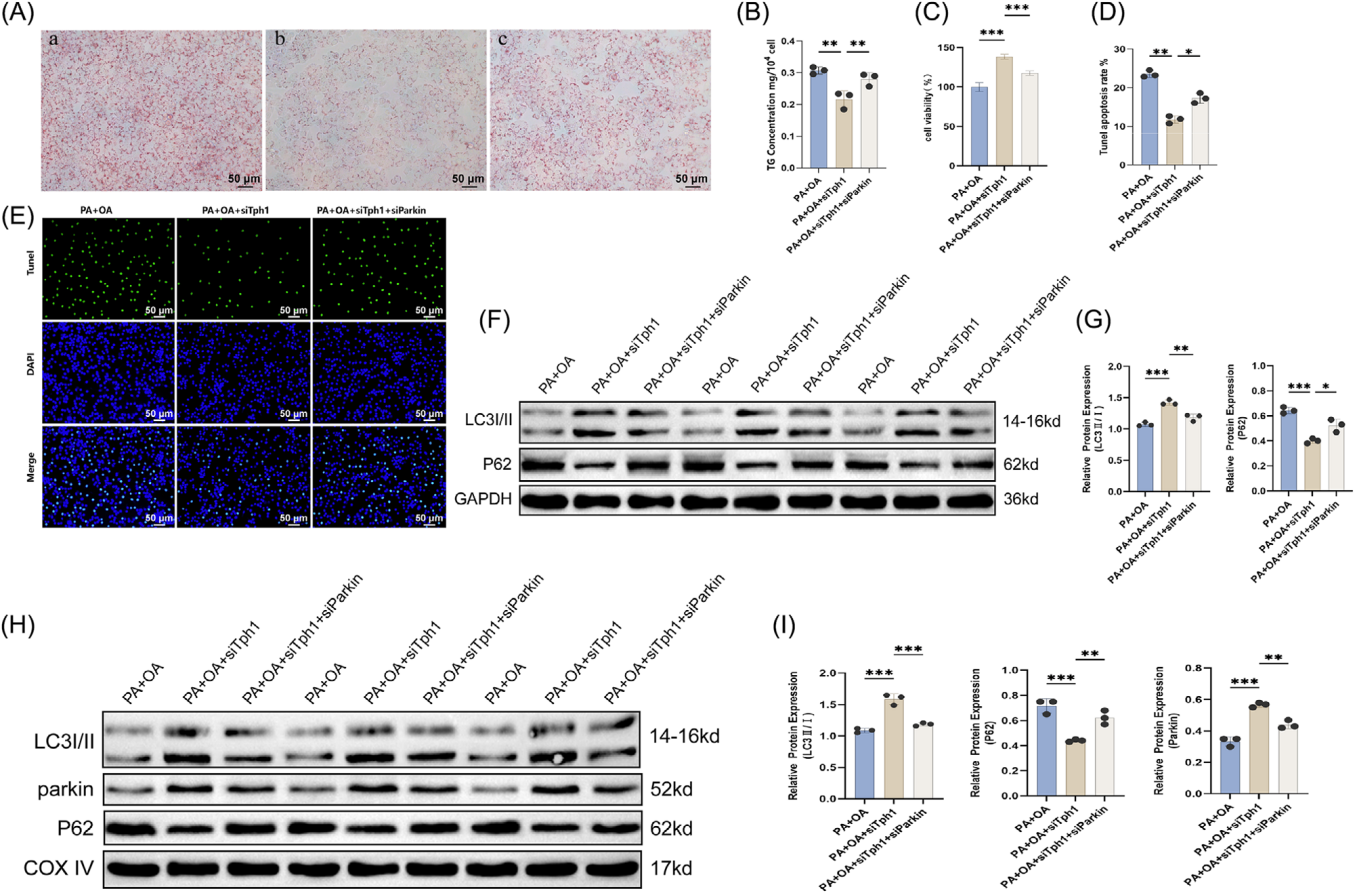
TPH1 regulated lipid accumulation and mitochondrial function through a Parkin‐dependent mitophagy pathway. (A) Oil Red O staining was performed to assess lipid accumulation in three groups: (a) PA + OA, (b) PA + OA + siTph1, and (c) PA + OA + siTph1 + siParkin. Scale bar = 50 µm. (B) Quantitative analysis of intracellular TG content (*n* = 3 per group). (C) Cell viability determined by MTT assay. (D) Quantification of apoptotic rates using TUNEL staining (*n* = 3 per group). (E) Representative TUNEL images of apoptotic cells. Scale bar = 50 µm. (F, G) Western blotting analysis and quantification of LC3 and p62 expression in total cell lysates (*n* = 3 per group). (H, I) Mitochondrial protein expression of LC3, Parkin, and p62 in each treatment group (*n* = 3 per group). Data are presented as mean ± SD. ^*^ *P* < 0.05; ^**^ *P* < 0.01; ^***^ *P* < 0.001. OA, oleic acid; PA, palmitic acid.

Parkin, a key regulator of PINK1‐dependent mitophagy, plays an essential protective role in the removal of damaged mitochondria during NAFLD progression.^25^ To determine whether *Tph1* contributes to MASLD pathogenesis through the Parkin‐mediated mitophagy pathway, we established PA + OA‐induced AML12 cell steatosis models and compared *Tph1* knockdown alone with combined *Tph1* and *Parkin* knockdown. Oil Red O staining showed that PA + OA significantly increased intracellular lipid droplet accumulation, whereas *Tph1* silencing markedly reduced lipid deposition. However, the protective effect was largely abolished when Parkin was co‐silenced, resulting in re‐accumulation of lipid droplets (Figure 8A). Similarly, TG measurements revealed parallel trends (Figure 8B), suggesting that the lipid‐lowering effects of *Tph1* inhibition depend on Parkin‐mediated downstream mechanisms. MTT assays showed that *Tph1* knockdown improved cell viability, while *Parkin* co‐silencing reversed this benefit (Figure 8C). TUNEL staining and quantitative analysis further demonstrated that *Tph1* knockdown reduced apoptosis, whereas dual knockdown of *Parkin* restored apoptotic levels (Figure 8D,E). To further assess the involvement of Parkin‐related mitophagy, we analyzed autophagy‐related protein expression in total and mitochondrial fractions. *Tph1* knockdown enhanced autophagic flux, evidenced by increased LC3‐II/I and decreased p62, whereas these effects were partially abolished by co‐silencing *Parkin* (Figure 8F,G). In mitochondrial fractions, *Tph1* silencing increased Parkin and LC3 levels and reduced p62, while the dual knockdown impaired these changes (Figure 8H,I).

Together, these results support an association between *Tph1* suppression and improved lipid metabolism and mitochondrial homeostasis in a Parkin‐dependent context. The partial loss of protection after Parkin co‐silencing suggests that mitophagy‐related alterations are an important downstream event in the observed phenotype. However, the present data do not establish the direct molecular mechanism by which TPH1 regulates Parkin, nor do they definitively verify true mitophagic flux activation.

## DISCUSSION

MASLD is increasingly recognized not only in adults but also in children and adolescents, paralleling the rising prevalence of pediatric obesity and metabolic syndrome. Early‐onset MASLD in children is particularly concerning because it may progress more rapidly to advanced liver disease and predispose individuals to long‐term metabolic and cardiovascular complications. The pathogenesis of MASLD is closely associated with genetic variations and aberrant gene expression. TPH1 is the rate‐limiting enzyme for 5‐HT synthesis and is predominantly expressed in the intestine, pineal gland, adipocytes, platelets, and immune system.^26^ 5‐HT is synthesized from tryptophan (TRP) through hydroxylation and decarboxylation reactions catalyzed by TPH.^26^ As a monoamine neurotransmitter that regulates both central and peripheral physiological processes, 5‐HT functions in modulating hepatic blood flow, metabolism, and inflammatory responses. Previous studies have shown that 5‐HT contributes to metabolic disturbances and plays an essential role in MASLD development.^20^, ^27^, ^28^ For example, in MASLD mouse models, intestinal TPH1 expression is markedly elevated, leading to increased peripheral 5‐HT levels, suggesting an important contribution of the TPH1/5‐HT axis to NAFLD pathogenesis.^29^ Pharmacological inhibition of peripheral 5‐HT synthesis or direct inhibition of TPH1 activity improves hepatic steatosis in animal models.^20^, ^27^, ^30^, ^31^ Mechanistically, elevated peripheral 5‐HT activates the hepatic 5‐hydroxytryptamine receptor 2A (5‐HT_2A_R)‐peroxisome proliferator‐activated receptor γ2 (PPARγ2) signaling pathway, promoting the transcription of lipogenic genes and subsequently enhancing triglyceride deposition and steatosis.^20^ Increasing evidence also indicates that hepatocytes express TPH1, and pathogenic stimuli such as glucocorticoids, hyperglycemia, or saturated fatty acids upregulate hepatic TPH1 and 5‐HT_2A_R expression, contributing to excessive intrahepatic 5‐HT synthesis. This aberrant activation of the hepatic 5‐HT system has been proposed as a pathological warning signal directly involved in MASLD onset.^22^, ^23^, ^32^ Although the precise role of TPH1 in MASLD remains incompletely understood, alterations in TPH1 activity profoundly influence 5‐HT synthesis and distribution, and given the broad regulatory actions of 5‐HT on hepatic hemodynamics, metabolism, and inflammation, a strong association between TPH1 and MASLD development is likely. Consistent with previous findings, our *in vivo* and *in vitro* experiments demonstrated that *Tph1* inhibition improved lipid metabolic abnormalities and attenuated liver injury. In line with established mechanisms, HFD‐induced MASLD mice exhibited obesity, dyslipidemia, and elevated serum aminotransferases, whereas *Tph1* knockdown markedly ameliorated these abnormalities and reduced hepatic steatosis, inflammation, and fibrosis. Taken together, these findings suggest that TPH1 is associated with multiple pathological processes in MASLD, and that *Tph1* inhibition may improve lipid dysregulation and liver injury, at least in part, through altered 5‐HT production. Interestingly, some studies have reported that supplementation with the 5‐HT precursor TRP alleviates experimental NAFLD in mice, possibly by stabilizing the upper‐intestinal epithelial barrier and modulating the dysregulated intestinal serotonin system, although the underlying mechanisms remain unclear.^33^ Importantly, because the present study was performed in young adult male mice and AML12 hepatocytes, its relevance to pediatric MASLD should be interpreted as translational rather than as direct clinical evidence in children, and potential age‐ and sex‐related differences remain undefined.

Impaired immune and inflammatory responses are central to MASLD progression. Lipid accumulation in the liver is accompanied by immune‐cell infiltration, resulting in low‐grade chronic hepatic inflammation. Adipose‐tissue expansion promotes the release of adipokines that influence hepatic lipid accumulation and immune‐cell recruitment.^34^ Macrophage infiltration into visceral adipose tissue accelerates insulin resistance, increasing fatty‐acid flux to the liver and enhancing *de novo* lipogenesis. When lipid accumulation exceeds hepatic metabolic capacity, lipotoxicity ensues, leading to oxidative stress, ER stress, inflammasome activation, and hepatocyte apoptosis. Apoptotic bodies further drive tissue regeneration and activate hepatic stellate cells (HSCs), promoting extracellular matrix deposition and fibrosis.^35^ TPH1 upregulation increases serum and hepatic 5‐HT levels.^36^ Excess 5‐HT promotes the release of pro‐inflammatory cytokines (e.g., IL‐6 and TNF‐α) from hepatic macrophages and other immune cells and activates oxidative‐stress pathways, exacerbating liver injury.^37^ Pang et al.^38^ demonstrated that *Tph1* knockout (5‐HT depletion) significantly reduced inflammatory cytokines and attenuated oxidative stress in Con A‐induced immune‐mediated liver injury. Moreover, 5‐HT depletion reduced hepatic fibrosis by inhibiting TGF‐β1/Smads signaling. In MASLD, activation of 5‐HT and its receptors similarly contributes to hepatic inflammation and fibrogenesis.^38^ 5‐HT acts synergistically with platelet‐derived growth factors to stimulate HSC proliferation and promote MASLD progression.^39^, ^40^, ^41^ Mechanistic studies show that activation of HSC 5‐HT_2A_R receptors triggers the 5‐HT/5‐HT_2A_R/PPARγ2 axis, upregulating pro‐inflammatory genes, promoting collagen deposition, and accelerating fibrogenesis; conversely, pharmacological blockade of 5‐HT_2A_R ameliorates hepatic inflammation and fibrosis.^20^ Organoid models also implicate 5‐HT_2A_R in FFA‐induced fibrosis in human liver spheroids.^27^ Moreover, 5‐HT acting on 5‐HT_2A_R and 5‐HT_2B_R drives HSC activation into myofibroblasts and triggers ROS accumulation, oxidative stress, inflammation, and fibrosis.^39^, ^42^, ^43^, ^44^ Consistent with these mechanisms, our findings demonstrated that *Tph1* inhibition significantly reduced hepatic oxidative stress, inflammation, and fibrosis. Compared with MASLD mice, *Tph1*‐suppressed mice exhibited increased SOD and GSH levels, decreased MDA production, reduced pro‐inflammatory cytokine release (TNF‐α, IL‐6), and attenuated HSC activation and collagen deposition (α‐SMA and Collagen I). These findings are consistent with previous reports on the pathological role of 5‐HT in liver injury, although the present study did not directly assess hepatic 5‐HT levels or 5‐HT_2A_R signaling activity.

Mitochondrial dysfunction and hepatocyte apoptosis are pivotal pathological events in the progression of MASLD and are widely regarded as the driving force behind the transition from simple steatosis to inflammation and fibrosis.^45^, ^46^ Substantial evidence indicates that lipid overload, lipotoxicity, and oxidative stress can induce mitochondrial membrane potential collapse, reduced ATP generation, and mtDNA damage, subsequently activating apoptotic pathways characterized by BAX upregulation, cytochrome‐c release, and caspase‐3 cleavage.^47^, ^48^, ^49^ Importantly, lipid deposition–induced lipotoxicity further contributes to mitochondrial impairment, ER stress, and oxidative stress in hepatocytes, thereby promoting the evolution of MASLD into MASH and ultimately cirrhosis.^50^, ^51^ Impairments in mitochondrial oxidative phosphorylation during MASLD disrupt energy homeostasis, diminish ATP production, and directly precipitate hepatocyte injury and apoptosis. Previous research has shown that 5‐HT exacerbates oxidative stress and mitochondrial toxicity in nonalcoholic steatohepatitis mouse models.^19^ Consistent with these reports, we observed marked mitochondrial dysfunction and enhanced apoptosis in MASLD mice, as evidenced by increased expression of c‐caspase‐3, BAX, and cytochrome‐c, along with a greater number of TUNEL‐positive cells. Concurrently, reductions in ATP content, mtDNA copy number, and JC‐1 red/green fluorescence ratio further indicated substantial mitochondrial deterioration. These findings align well with established pathogenic paradigms, reinforcing mitochondrial injury and apoptosis as central drivers of MASLD‐related liver damage. Notably, liver‐specific suppression of TPH1 significantly alleviated these pathological changes in our study, suggesting that TPH1 upregulation may actively contribute to hepatocyte injury in MASLD. Previous studies have implicated the TPH1/5‐HT axis in the regulation of lipotoxicity, inflammation, and metabolic imbalance. Mechanistically, 5‐HT acting through 5‐HT_2A_R has been shown to aggravate mitochondrial oxidative stress, disrupt calcium homeostasis, and activate pro‐apoptotic signaling, thereby accelerating hepatocyte injury.^20^ In line with this mechanism, *Tph1* knockdown in our study led to marked restoration of ATP levels, improved mitochondrial membrane potential, increased mtDNA copy number, and reduced expression of BAX, c‐caspase‐3, and cyt‐c, accompanied by fewer TUNEL‐positive cells. These findings suggest that TPH1 may be involved in MASLD progression by promoting mitochondrial deterioration and apoptosis, but the relative contribution of reduced 5‐HT signaling cannot be determined from the present data.

Parkin activation plays a crucial role in maintaining mitochondrial integrity by mediating the selective removal of damaged mitochondria, thereby preserving cellular homeostasis. PINK1/Parkin‐dependent mitophagy has been demonstrated to exert hepatoprotective effects in MASLD by promoting mitochondrial quality control, reducing oxidative stress and inflammation, and improving lipid metabolic balance.^52^, ^53^ Conversely, Parkin deficiency leads to the accumulation of dysfunctional mitochondria and aggravates steatosis, inflammation, and fibrosis.^54^ Previous studies have indicated that 5‐HT suppresses autophagy by activating the mammalian target of rapamycin (mTOR) pathway, thereby impairing mitochondrial function and promoting hepatocellular injury.^55^ Pharmacological inhibition of TPH1 has been reported to reduce mTOR signaling, enhance autophagic activity, and facilitate the clearance of damaged mitochondria.^55^ In MASLD mouse models, *Tph1* inhibition significantly restores mitophagy and reduces hepatic lipid accumulation and oxidative stress.^27^ Thus, reducing 5‐HT levels may help restore autophagy, alleviate oxidative stress, and enhance PINK1/Parkin‐mediated mitophagy, collectively protecting hepatocytes from lipotoxic injury. In our study, *Tph1* inhibition was associated with changes consistent with enhanced Parkin‐mediated mitophagy. TEM analyses revealed reduced mitochondrial swelling, improved cristae morphology, and an increased number of mitophagosome‐like structures. Concurrently, mitochondrial Parkin enrichment was elevated, accompanied by increased LC3‐II levels and reduced p62 accumulation, findings compatible with altered mitophagy‐related activity. However, because autophagic flux blockade assays were not performed, these data did not definitively confirm true mitophagic flux activation. These findings support an association between *Tph1* suppression and improved mitochondrial quality control in MASLD, but do not establish whether this effect is directly mediated by TPH1 or indirectly related to altered serotonin signaling. In addition, the direct molecular mechanism linking TPH1 to Parkin remains unclear, because we did not investigate whether TPH1 affects Parkin expression, mitochondrial translocation, protein stability, or upstream PINK1 signaling.

Although our study provides strong evidence for a pathogenic role of hepatic TPH1 in MASLD, several limitations warrant careful interpretation. Traditionally, TPH1 is recognized as a peripheral enzyme primarily expressed in enterochromaffin cells, and the liver is not considered a principal site of 5‐HT synthesis. However, emerging studies have demonstrated that hepatocytes can upregulate TPH1 and 5‐HT_2A_R expression under metabolic stress conditions such as hyperglycemia, glucocorticoid exposure, or elevated saturated fatty acids, accompanied by increased intrahepatic 5‐HT production that drives lipid accumulation, inflammation, and tissue injury.^22^, ^23^, ^32^ This indicates that hepatic TPH1 may acquire the capacity to promote local 5‐HT synthesis in pathological states. Based on this, the hepatoprotective effects of *Tph1* knockdown observed in our study may partially result from reduced hepatic 5‐HT production and weakened 5‐HT signaling, rather than reflecting 5‐HT‐independent functions of TPH1. Notably, we did not measure intrahepatic 5‐HT levels or assess 5‐HT_2A_R signaling activity following *Tph1* suppression, making it difficult to rule out the possibility that the beneficial effects were mediated predominantly by altered 5‐HT metabolism. Therefore, our study does not permit a definitive distinction between direct effects of TPH1 and secondary effects mediated by the hepatic 5‐HT axis. In addition, the present study was performed in young adult male C57BL/6J mice and AML12 hepatocytes, without pediatric human samples, juvenile animal models, or sex‐comparative analyses. Therefore, its relevance to pediatric MASLD should be interpreted as translational rather than direct clinical evidence, and potential age‐ and sex‐related differences remain to be clarified. Furthermore, although Parkin co‐silencing partially reversed the protective effects of *Tph1* knockdown, our study did not determine the direct upstream mechanism by which TPH1 influences Parkin‐related mitophagy. Specifically, we did not assess whether TPH1 regulates Parkin at the transcriptional, post‐transcriptional, or mitochondrial recruitment level. In addition, mitophagy in the present study was evaluated mainly by TEM and protein markers, without lysosomal blockade or other autophagic flux assays. Therefore, we cannot definitively distinguish true mitophagy activation from altered autophagic flux. Moreover, our study does not clarify whether TPH1 acts independently of 5‐HT to regulate mitochondrial quality control, autophagy, or apoptosis, and these mechanisms must be explored in future work. Additional limitations include the use of siRNA‐mediated gene silencing, which reflects short‐term effects but does not model chronic metabolic disease progression, and an inability to distinguish the relative contributions of hepatic vs. gut‐derived 5‐HT. Given the low basal expression of hepatic TPH1, the factors driving its pathological upregulation, whether inflammatory cytokines, metabolic stress, or gut‐liver axis feedback, remain unclear. Future research should incorporate pediatric clinical samples, age‐appropriate models, and sex‐based analyses, together with hepatocyte‐specific *Tph1* knockout models, direct quantification of hepatic 5‐HT, 5‐HT_2A_R inhibition studies, and detailed analysis of the upstream events governing Parkin activation, as well as formal autophagic flux assays, to further define the causal role and translational relevance of TPH1 in MASLD.

In conclusion, this study is the first to identify hepatic TPH1 as a potential regulator of mitochondrial quality control during the development of MASLD. We demonstrated that TPH1 expression is markedly upregulated in MASLD, whereas selective inhibition of hepatic TPH1 effectively attenuates hepatocellular lipid accumulation, apoptosis, and mitochondrial dysfunction. *Tph1* suppression was associated with mitophagy‐related improvements, restored mitochondrial membrane potential, increased ATP production, and preserved mtDNA integrity, thereby alleviating hepatocellular injury. These findings suggest that TPH1 is involved in MASLD progression and is associated with impaired mitophagy. However, because hepatic 5‐HT levels and 5‐HT_2A_R signaling were not directly assessed, whether the observed effects are direct or mediated by altered serotonin signaling remains to be determined. Therefore, hepatic TPH1 should be regarded as a candidate therapeutic target in experimental MASLD, and its precise mechanistic and translational relevance requires further validation.

## CONFLICT OF INTEREST

The authors declare no conflict of interest.

## Supporting information



Supporting Information
