# Supplementary material for: The role of tryptophan hydroxylase‐1 in metabolic dysfunction‐associated steatotic liver disease
Source: Pediatr Investig. 2026 Aug 31:10.1002/ped4.70078. Online ahead of print. doi: 10.1002/ped4.70078 (PMC13529607; doi:10.1002/ped4.70078)
Supplement: Supplementary file 1 [file PED4-9999-0-s001.pdf]

Supplementary Material for

The role of TPH1 in metabolic dysfunction-associated steatotic liver disease: mechanistic insights with potential relevance to pediatric MASLD

Shuangzhen Jia, Xiaolin Ye, Zhaoxia Wang, Jie Wu

Table S1. The sequences of siRNAs

siRNA	Sense strand (5′→3′)	Antisense strand (5′→3′)
siTph1	5′-GGCUUGCUUUCUUCCAUCAGUU-3′	5′-CUGAUGGAAAGAAAGCAAGCCUU-3′
siTph1 NC	5′-CCUCCAAGGAAACCAUCAAUU-3′	5′-ACGUGACACGUUCGGAGAAUU-3′
siParkin	5′-CCUCCAAGGAAACCAUCAAUU-3′	5′-UUGAUGGUUUCCUUGGAGGUU-3′

Table S2. List of primers used for qRT-PCR

Gene	Forward primer sequences	Reverse primer sequences
Actb	5'-CATGTACGTTGCTATCCAGGC-3'	5'-CTCCTTAATGTCACGCACGAT-3'
Tph1	5'-TTCTGACCTGGACCATTGTG-3'	5'-CTCTCTGCAAGCATGGGTT -3'

Table S3. List of primers used for qRT-PCR

Gene	Forward primer sequences	Reverse primer sequences
Actb	5'-GTCCCTCACCCTCCCAAAAG-3'	5'-GCTGCCTCAACACCTCAACCC-3'
mtDNA	5'-GCCGGTGACTACGACTGAA-3'	5'-CACTGGCCTGCAAGTCTTC -3'

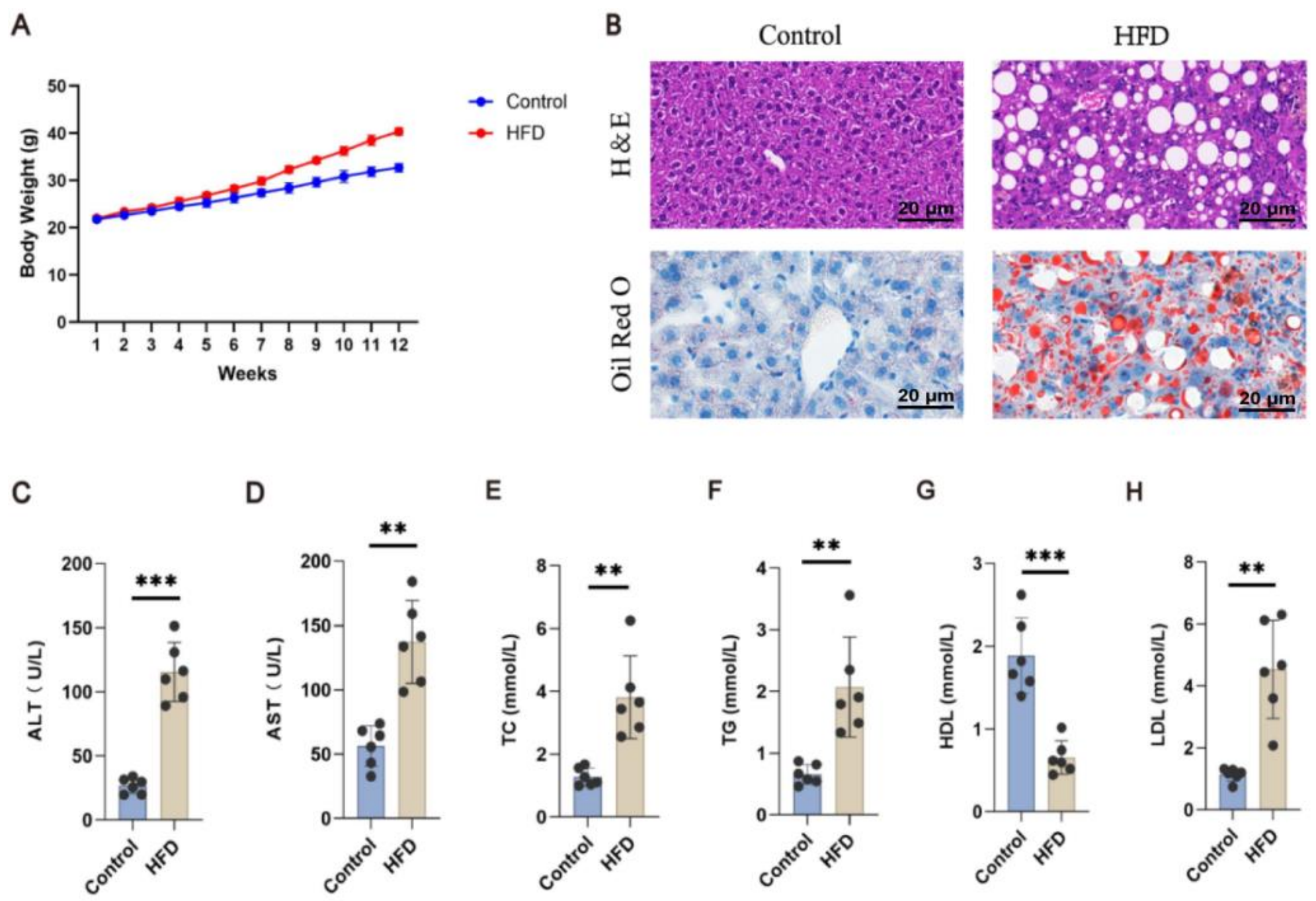

Figure S1. Establishment and validation of the MASLD mouse model. (A) Time-course of body weight over 12 weeks in Control vs. HFD-fed mice. (B) Representative liver histology: H&E staining shows hepatocellular swelling, vacuolar degeneration, and abundant lipid accumulation; Oil Red O staining confirms diffuse neutral lipid deposition. Scale bar = 20 μm. (C–H) The levels of ALT, AST, TG, TC, LDL-C and HDL-C in the serum (*n* = 6 per group). Data are presented as mean ± SD. $^{*}P < 0.05$; $^{**}P < 0.01$; $^{***}P < 0.001$. MASLD, metabolic dysfunction-associated steatotic liver disease; TG, triglycerides; TC, total cholesterol; LDL, low-density lipoprotein cholesterol; HDL, high-density lipoprotein cholesterol; AST, aspartate aminotransferase; ALT, alanine aminotransferase; HFD, high-fat diet.
